# Effect of social relationships on antiretroviral medication adherence for people living with HIV and substance use disorders and transitioning from prison

**DOI:** 10.1186/s40352-015-0030-6

**Published:** 2015-12-18

**Authors:** Julia Rozanova, Shan-Estelle Brown, Ambika Bhushan, Ruthanne Marcus, Frederick L. Altice

**Affiliations:** 1 Department of Internal Medicine, Section of Infectious Diseases, AIDS Program, Yale University School of Medicine, 135 College Street, Suite 323, New Haven, CT 06510-2283 USA; 2 Division of Epidemiology of Microbial Diseases, Yale University School of Public Health, New Haven, CT USA; 3 Harvard Medical School, Boston, MA USA

**Keywords:** People living with HIV, Addiction, Prisoners, Adherence, Antiretroviral therapy, Social relationships, Family, Friends, Clinicians, Qualitative interviews, Trust in Physician

## Abstract

**Background:**

This paper examines how family and social relations facilitate and inhibit adherence to antiretroviraltherapy (ART) for people living with HIV (PLH) who have underlying substance use disorders and are transitioningto the community post-incarceration.

**Methods:**

Combining the methods of inductive close reading and constantcomparison, we analyzed the data from 30 semi-structured interviews of PLH who had recently transitioned to thecommunity within the previous 90 days.

**Results:**

Three central themes were anticipated as important socialrelationships post-release: self-reported family, friends and clinicians. Among these, four sub-themes (social isolation, ‘double jeopardy’, search for belonging, and trust and respect) emerged, highlighting how they impacted ART adherence. Post-release, participants returned to resource-poor communities where they experienced socialisolation. ART adherence was enabled by having a purpose in life, which correlated with having robust family support structures. Many former prisoners felt that a chasm between them and their families existed, both because of HIV stigma and their addiction problems. In this context, relationships with untrustworthy friends from their druguse networks led to relapse of drug use and risky behaviors, jeopardizing participants’ ART adherence and persistence. To avoid the double jeopardy, defined as seeking friends for support but who were also the ones who contributed to drug relapse, participants searched for new social anchors, which often included their healthcare providers who represented trusted and respected persons in their life.

**Conclusions:**

While some former prisonersperceived doctors as uncaring and their relationships asymmetrical, positive relationships with these providers,when respect and trust was mutual, reinforced the participants’ sense of belonging to what they called ‘the world that don’t do drugs’ and motivated them to adhere to ART.

## Background

Antiretroviral therapy (ART) adherence (i.e., taking medications as prescribed) and persistence (i.e., remaining on medications continuously) (Bae et al. 2011) is crucial to society and people living with HIV (PLH) who transition through prisons and jails. Consequently, ART adherence and persistence remains necessary for ‘Treatment as Prevention’ to work. With 2.2 million people incarcerated in the U.S. and 12 million transitioning through these facilities annually, criminal justice involvement disrupts the lives of as many as 1 in 6 PLH (Spaulding et al. 2009). It is well-documented that most PLH in prisons or jails initiate ART while incarcerated and have high levels of ART adherence and viral suppression while there (Meyer, Cepeda, Springer, et al. 2014; Springer et al. 2004; Substance Abuse and Mental Health Services Administration (SAMHSA) 2009). These benefits, however, are often not sustained for nearly three-fourths of those prescribed ART, as they either become suboptimally adherent with their medications or discontinue them completely within 3 months post-release (Meyer et al. 2014; Springer et al. 2004). Now that guidelines recommend ART for all PLH, regardless of CD4 cell count, new and invigorated efforts are essential to understand the transitional process for PLH who re-enter the community after incarceration.

Numerous factors impede ART adherence in former prisoners with substance use disorders (SUDs). These include organizational and bureaucratic barriers to care (Holtzman et al. 2015), reintegration activities as competing demands to accessing care (Sidibe et al. 2015a), poverty and economic insecurity (Harding et al. 2014), lack of health insurance and secure housing (Chen et al. 2013; Zelenev et al. 2013), lack of addiction treatment services and challenges with coordinating treatment for multiple comorbidities like mental illness, HCV and other chronic medical conditions, especially as PLH age (Althoff et al. 2013; Springer et al. 2011), and relapse to drug use (Binswanger et al. 2011). In New Haven, factors impeding connection of former prisoners to HIV care are exacerbated by the context of New Haven being the 7^th^ poorest city for its size and high socio-economic disparities with one third of inhabitants living below the 2011 Federal poverty level (compared to 9 % statewide). Experiences of post-incarceration transitions for former prisoners in New Haven are also shaped by higher racial disparities in incarceration rates between black and white residents in CT than nationwide (Copollo and McCarthy 2008) and harsher mandatory minimum prison-term penalties for non-violent drug offences (De Avila 2015).

Central to improving ART adherence in this vulnerable population is reducing relapse to drug and alcohol use (Kamarulzaman and Altice 2015). Social support from family, friends, and community enables retention in care (Holtzman et al. 2015), reduces risk of relapse, and alongside high interpersonal trust and civic participation, forms social capital (Berkman 2000) which is a major determinant of health (Kawachi et al. 2004). Research within prison settings suggest that trust positively influences ART acceptance and adherence (Altice et al. 2001; Mostashari et al. 1998), and healthcare utilization among people who inject drugs (PWID) (Ostertag et al, 2006). The term ‘trust in respected others’ was offered to more broadly include elements of trust beyond the physician (Hammett et al. 2015b; Ostertag et al. 2006; Sidibe et al. 2015b), including bi-directional relationships (such as with the healthcare provider) that promotes better health outcomes. HIV-infected criminal justice populations, however, may have little contact with family and/or have non-supportive family relationships, and thus lack these sources of social support (Meyer et al. 2013). They may search for respected and trustworthy connections with healthcare linkage specialists (either peers or professionals) who have been shown to promote patient motivation to better engage in care (Broaddus et al. 2015). Peer relationships may conversely place PLH in jeopardy if peers endorse risk behaviors: social network research suggests spreading of such behaviors through social contagion (Christakis and Fowler 2013). As PLH are often socially isolated and have fewer opportunities than other individuals (Phillips 2011), it is crucial to examine when and how social relationships may alternatively serve as potential barriers or facilitators to ART adherence and retention in care. In line with the goal of developing social environments that bolster formerly incarcerated individuals’ ability to manage living with HIV (Auerbach 2009), this paper examines, using a qualitative lens, how social and family relationships influence ART adherence among PLH who recently transitioned from prison to the community.

Qualitative inquiries add contextual understanding to extant quantitative literature about post-release transition from correctional facilities to the community (Iroh et al. 2015; Morozova et al. 2013; Springer et al. 2011), specifically knowledge about the lived reality of these persons’ experiences with family, friends and healthcare providers (Hammett et al. 2015b; Sidibe et al. 2015b), and examines exactly how and why they experience real-life exigencies with adhering to ART after release from prison. How to optimize ART adherence and persistence is crucial for incarcerated PLH already engaged in care to achieve optimal results from the individual and public health perspectives. Revealing relationship-connected factors that shape adherence among those already in care is also important to provide new insights on issues faced by those who are not engaged, but nonetheless need it.

## Methods

### In-depth Interviews

We used data from 30 qualitative interviews with PLH who were released from prison or jail within the previous three months. A convenience sample of participants was recruited from two large quantitative studies that examined health behaviors and linkage to care among PLH who were transitioning to care in New Haven, Connecticut (Bhushan et al. 2015). One purpose of the in-depth qualitative interviews was to understand what could serve as barriers and as facilitators for taking ART in the context of transition from prison to community. The interview guide included open-ended questions based on extant literature and was developed to understand better how transition shaped social recovery, specifically re-engaging of ex-prisoners with the community and with the social roles they find meaningful (Pelletier et al. 2015), and ART use for recently released PLH. One of the open-ended questions involved how they were managing their ART currently and if they described taking it every day as prescribed, this was recorded as being adherent; objective measures were not used to define adherence. All interviews were conducted in late 2011 in private settings by one of the authors (AB) and lasted 45 – 90 minutes; only one participant was re-incarcerated and was interviewed in jail. Interviews were audio-recorded and transcribed verbatim. The study was approved by Yale’s Institutional Review Board.

### Study population

Eligibility criteria for participation included only PLH who had been released from prison or jail in the previous 3 months. All participants, consistent with the HIV epidemic in criminal justice populations in Connecticut, had underlying SUDs, mostly with related risk for HIV being past or current drug injection (Meyer et al. 2014; Meyer et al. 2014). Participants were thus documented as being HIV-infected with an underlying substance use disorder (i.e. participants self-reported having used drugs in the past and/or trying to stay clean), and were returning to communities in Connecticut, many of which had high rates of concentrated poverty with up to 48 % of the population living below the poverty line (Buchanan and Abraham 2015). All participants were linked to Ryan White-subsidized HIV care and ART through the parent studies, were aware of having HIV, and had been prescribed ART during their most recent incarceration.

### Analysis

Following the principles of narrative analysis of text (Denzin and Lincoln 2008), interview transcripts were first closely and repeatedly read to familiarize authors with participants’ experiences with community transition, ART adherence, and social relationships. We used the ‘comment box’ function in Word to highlight cogent quotes. One co-author (JR) coded the interview transcripts using NVivo9 qualitative data management software (QSR International Pty Ltd. Version 9, 2010). The coding structure was initially developed by two co-authors (AB and JR) to include both anticipated and emergent themes and reviewed and refined by all co-authors to ensure agreement. This paper is based on the detailed analysis of how family and social relationships shape ex-prisoners’ adherence to ART. We took a qualitative interpretive approach, combining the methods of inductive close reading and constant comparison (Green and Thorogood 2004; Gubrium and Holstein 2009) to examine subthemes that concerned the role of social and family relationships on ART adherence . Each transcript was read line by line by the coder (JR) to identify sub-themes. Bi-weekly discussions among all co-authors served to review the coding as it progressed and ensure inter-rater reliability through discussion of any disagreement. During these meetings we identified and analyzed in more detail four emergent sub-themes that highlighted both positive and negative perceptions of social relationships and their impact: (1) social isolation; (2) trust and respect; (3) double jeopardy of relationships; and (4) search for social anchors and belonging. These sub-themes were present across all identified relationships that helped facilitate or impede ART adherence, namely: participants’ relationships with family, friends, and clinicians. While the interviews asked about family, friends, and clinicians, the questions did not impose a definition. Consequently, participants were allowed to define who was considered as family, friends, or clinicians. For example, some participants described clinicians whom they greatly appreciated as being almost like family. Conversely, others stated that indifferent parents or siblings ceased being their family while others explained their family included only their spouse and children. Similar nuances emerged with how some participants defined ‘friends’. Below we discuss the results of our analyses, presenting how the three kinds of participants’ relationships facilitated or hindered ART adherence during transition from jail/prison to the community.

## Results

Participants’ (*N* = 30) characteristics are described in Table 1 with the majority being marginally housed, few were married, and only one was employed.

**Table 1:** Participants' characteristics

Participants’ characteristics		N
Mean Age (years)	50 (SD 11.7)	
Mean time since HIV diagnosis (years)	17.6 (SD 8.6)	
Sex	Male	25 (83 %)
	Female	5 (17 %)
Race	Black	17 (57 %)
	Hispanic	10 (33 %)
	White	3 (10 %)
Housing situation	Halfway house/transitional HIV housing shelter	11 (37 %)
	Homeless	8 (27 %)
	Living with friends or family	6 (20 %)
	Own or rent house/apartment	4 (13 %)
	Re-incarcerated	1 (3 %)
Source of livelihood	Employment	1 (3 %)
	Social security	25 (83 %)
	None	4 (13 %)
Marital status	Married	3 (10 %)
	Single	14 (47 %)
	Divorced/separated	13 (43 %)
Has been incarcerated >1 time		29 (97 %)

ART adherence was supported by a sense of belonging and having a purpose in life, which sometimes stemmed from having robust family support structures. Participants who had close positive relationships with family wished to stay well and alive for the sake of loved ones, which promoted ART adherence. Many ex-prisoners, however, felt that they had permanently severed any meaningful relationships with their families because of their addiction problems and discrimination from HIV. In this context, social relationships with friends from their drug-use networks made participants more vulnerable to relapse to risky behaviors, leading to non-adherence and non-persistence of ART use. To escape this jeopardy, participants searched for other social anchors. Often, their closest social relationships were with their HIV care providers. While some ex-prisoners perceived doctors as uncaring and patriarchal, positive perceptions of relationships with these providers reinforced the participants’ trust and sense of belonging to what they called *‘the world that don’t do drugs’* and motivated them to adhere to ART. Below we present these findings, illustrating them with the participants’ quotes.

### Family: support facilitates but ostracism hinders ART adherence

Participants in our study varied in terms of their family backgrounds and the nature of their relationship with their family. Some participants took pride in growing up in stable households that Anderson (1999) described by the folk term ‘decent’ that had no involvement with the criminal justice system, saying:

*None of my family has been in trouble AT ALL.*

*I grew up in a good family. And I’d never been in trouble with the law.*

Such households were often headed by a respected female figure like a mother or a grandmother. This is illustrated in the following anecdote where the death of this figure disrupted a family’s ties and togetherness:

*We all used to be so close. And my grandmother dies and everything gets out of whack and falls apart. She was the whole to our family for everything.*

Participants who came from intact families stressed the importance of love and support from their family in ensuring a smooth post-prison transition:

*I didn’t really have no problem transitioning [from prison to the community] ‘cause I got [a] pretty big family. If I ask them, they’ll help me. I got people that love me.*

Families also anchored participants in the present and gave them a sense of purpose for the future and a goal to live for, which motivated them to adhere to ART:

*For the last 20 years, I’ve been okay ’cause I take care of myself. I know I need this [ART medication] and I have a family. I don’t want to die. So I do take care.*

*I can overcome [HIV], the drugs, because I got a lot of people that loves me. I got my family supporting me. I got my wife supporting me. I got my kids supporting me. Everybody is counting on me. So all eyes are on me.*

*My wife, she make sure I go to doctor’s appointments.*

But family ties were not supportive of all participants. Family members often struggled with the same issues as the participants themselves, and had few resources or agency to provide support to others. In fact, many participants spoke about family dysfunction and the poverty, violence or drug use they witnessed or experienced from a young age:

*I grew up in a housing project. So, at nine years old, I tried marijuana and I liked it.*

*I have a sister that passed away. Her boyfriend was an intravenous drug user. And he died of AIDS, and she did too.*

When their family members either died or themselves struggled with crippling substance use disorders or other co-morbid conditions, these participants could not count on them for emotional or instrumental support to keep their appointments and adhere to their ART. As all our participants had a history of substance use disorders, they frequently noted how their family ostracized them:

*I have a sister here in this town. I see her passing and we’ll have a five-minute conversation. But I'm not allowed to go to her house. I don’t know her phone number.*

Another participant shared that

*Drugs ruined my life. My friends and family lost respect for me.*

And yet another one stated that

*They're [his parents] somewhere either in Alabama or Florida. I haven't seen them in over 20 years. Twenty years ago, I needed help. “Don’t call here.” And their phone number was changed.*

While drug addiction may have started participants’ alienation from family, receiving their HIV diagnosis deepened this divide and made family support more tenuous. Participants often self-isolated, out of internalized HIV stigma and fear and shame reinforced by the process of receiving care:

*You have to go there every day and you have to get blood tests every time. And that’s not normal. It made me stay away from my family. Because I am HIV+ and they’re not, I just push myself away. I still call my dad, talk to him, but I haven't seen them in years.*

In sum, family support could be a powerful motivator for ART adherence for those who had intact and robust family networks. Many individuals, however,  lacked family support either due to membership in families that had chronic medical illnesses or substance use disorders and therefore were ill-equipped to provide material and psychological support. This, in turn, led to self-isolation or ostracization from their families due to their own criminal, drug use or HIV histories.

### Friends: support by respected friends enabled recovery while interactions with drug-using friends facilitated drug-use relapse

Individuals who had poor family support relied to a greater degree on their friendships. When relationships with friends were functional (i.e., supportive), this gave participants emotional support to overcome addictions and instrumental support with post-incarceration transition. As participants noted, this helped reduce their risk behaviors, avoided relapse to drug-use, and stabilized their life:

*A cellie - a friend of mine that’s my best friend -- she said, “Start reading the Bible.”And ever since I read it every day. I truly believe reading the Bible helped me to stay clean.*

*I didn’t have a place to stay, when I was in the halfway house. So, I have a friend that’s HIV positive. He is a gay guy, and he's a very, very good friend of mine. And he told me about resources he knew about.*

Longer-term friendships were also valued and while not directly instrumental to health-seeking, gave participants a sense of continuity and social belonging:

*All my friends that I grew up with and played football or baseball, or basketball with, once a year we have a reunion, and that’s when I see everyone.*

Not all participants had trustworthy friends willing to help them along their transitional journey. Additionally, even these valued friends had similar vulnerabilities as the study participants regarding health problems and criminal justice involvement. Participants longed to have reliable friends who would care about them and could provide support. Instead, many participants experienced social isolation, noting that friends did not write or visit them while they were incarcerated, and ignored them after release:

*[I was in jail] two and a half years. So nobody wrote me.*

*What I can consider a friend, I wouldn’t be staying in the shelter, right now. “Come on, [name]. Come stay on my couch ‘til you get on your feet.” Or, “I ain’t got no room , but you can make your pallet over there.” “You ain’t gotta be in the street.” No, I don’t have any good friends.*

There was also a broader sense of social marginalization that individuals experienced beyond their individual friendships. In the same way that many individuals noted being alienated from their families, participants felt excluded from their social communities due to stigma related to HIV:

*When I first found out that I had it, they used to whisper: “She is HIV. Don’t touch her.”*

From the vantage point of their experiences of illegal activities, addiction, and involvement with the criminal justice system, participants said that:

*There are two worlds. The world that do drugs and the world that don’t do drugs.*

Participants saw themselves living in a different world from people who did not use drugs, from which they felt excluded and disengaged. They furthermore felt unworthy of the company of individuals from this perceived alternate world, a notion that affected their self-esteem and feeling of acceptance. As these participants stated,

*I’ll never be somebody…I’ll never be good.*

*All the people that doing good, have a job, eating, drinking a beer a week. You can't find those people to socialize with because they don’t want to socialize with you ’cause they are thinking different, better, and they will not go down to that level.*

While feeling ignored or self-excluded from *‘the world that don’t do drugs’*, soon after release participants encountered multiple opportunities to reconnect to *‘the world that do drugs’*—the world from which many originally felt they hailed—which was a pathway to drug relapse and non-adherence to ART. Participants shared that upon release, their *‘street friends’* easily offered them drug credit, suggesting they could pay them back later. Given the chronic and recurring nature of addiction, such offers were hard to resist, and given the participants’ social isolation and other unmet needs like housing and employment, many reported getting re-involved with friends from *‘the world that do drugs’*, despite recognizing that these relationships were toxic and harmful to their goals of addressing their substance use disorders, managing their HIV, and avoiding re-incarceration. In contrast to true and respected friends, participants called these friends in risky behaviors ‘associates’:

*The only friends I have are my friends when I buy drugs or drink. So is that a friend? No, that’s an associate.*

*A friend of mine charges me $50 a week [for room]. It's in the heart of crack city. [It helps trigger] there’s no doubt. It's all around me. It's in my living room.*

*If I get with the wrong crowd, for days I’d be up running, ripping, hanging out. So I watch who my friends is now because they don’t care. As long as you spending money, they gonna party with you. They ain’t gonna say, “Oh, yo, yo, check yourself, stop for a minute. Yo, I like you a lot as a good friend. You need to take your meds”.*

### Clinicians: positive symmetrical relationships promoted ART adherence but dismissiveness undermined trust

Oftentimes, participants reported experiences of feeling uncared for, ignored, and just not taken seriously by some clinicians they had encountered. They were frustrated by asymmetrical relationships where they as patients felt excluded from decisions and ‘acted upon’ by their providers. Encounters with dismissive medical staff reinforced participants’ suspicion and mistrust in clinicians, whom they regarded as heralds of bad news. Participants resented when their questions to health-care providers went unanswered and their reaction was to withdraw, or even avoid these professionals altogether:

*They took my blood. I want to know why they took the blood. I asked them. They gave me some little skinny piece of paper that said they took this, this, this, this, and this. And then they circled this thing, and nobody tells me what the circle’s for. I got half answers.*

*I don’t like doctors period. This is my first time dealing with anything in a white coat. I don’t want to hear what he have to say. ‘Cause I already have too much to carry for me to hear another bad news.*

Not all clinicians, however, were painted with this same brushstroke. Participants distinguished between ‘caring’ and ‘uncaring’ clinicians, both within the prison medical system and in the community:

*There’s some staff that honestly care about the well-being of a person. And there some that just don’t care. “You're just here to be warehoused. Get out of my way. I'm just here to do my eight hours”.*

*Some doctors, I think, try to talk to you, include you in to whatever they’re trying to show you to help you live longer. Some doctors not. They just want you in and out.*

Oftentimes, a positive encounter with a ‘caring’ clinician who treated the patient with respect happened in the context of incarceration, was associated with HIV diagnosis and treatment, and turned into a long-standing relationship. In the context of such symmetrical relationships, participants trusted their doctors, felt positively about shared-decision making and ownership, and felt accepted and safe to be honest about their vulnerabilities, including their high risk behaviors, all of which were ultimately important for their adherence to ART:

*[My] doctor very clearly explains things that he’s doing to [me]. We have a good relationship. They sent him to me almost eight years now, and we just clicked. He likes me. I like him. I'm honest. I don’t play games. If I'm doing something, I’ll tell him. I'm not gonna lie to him.*

A key element in positive symmetrical patient-doctor relationships was that by including patients in treatment decisions, doctors created safe spaces of mutual trust where patients felt empowered to engage with information about their illness and make decisions about their healthcare:

*I try to participate in my treatment. Stay educated, ask questions. I been reading [on HIV] for years. I feel comfortable with them [the nurse and the doctor].*

Health then became a teamwork goal, as in this situation, where a participant spoke with pride of the validation she received from her doctor for showing steady continuous progress towards recovery:

*And [the doctor] told me I was the most-improved HIV patient he’s ever worked with. Like, he’s never seen their health bounce back so fast. And I'm very excited about that.*

Certainly, relationships with clinicians included a degree of performance and what Anderson (1999) called ‘gloss’, which facilitated smooth social interactions at the level of small talk pleasantries, and participants were realistic about it:

*I'm a people person. I got along with them doctors.*

Also participants described how doctors occasionally put pressure on patients regarding their drug use and made prescription of ART conditional on their non-relapse. This obvious display of the doctors’ power could cause anger, a sense of humiliation, and abandoning treatment, unless the patient respected the doctor unequivocally and trusted the doctor was acting in his best interests:

*I got this doctor for 10 years. He was my doctor in jail, in ’96. And he told me, “If you’re going back to the community and you’re doing drugs, I don’t give you no medication.” Well, [I changed] for him ’cause he is a good doctor, you know what I mean?*

Participants who, as described earlier, perceived they had a deficit of credibility and often lacked supportive relationships with family and friends, cherished positive relationships with doctors, which gave a sense of belonging and bridging to ‘*the world that don’t do drugs’*, enabling participants’ motivation to live, giving them hope, and making adherence to ART meaningful:

*Because I want to live, I know I have to take the medication. It's simple.*

*I'm starting to take it [ART] more serious ‘cause I want to live a little longer anyway.*

*Managing my HIV is, basically, just taking your meds, doing what your doctor tells you to. And I do that faithfully. I don’t miss a day. Never miss a day.*

Indeed, in describing relationships with caring doctors, participants invoked the concepts of family and friends, meaning that they were treated by doctors as their own kin, and signifying that participants felt accepted, supported and cared for on a profoundly human level:

*I like going to see Dr. X, I look at him not just a doctor, as a friend.*

*I have my adoptive mother, Dr. Y, she's the chief psychiatrist of [hospital]. When I was in jail, she sent $50 every month. And she’s been like a mother to me. Even to this day, once a month she comes up here, and visits me. She's just been very supportive of me.*

Even supportive symmetrical relationships with clinicians had to endure strain. With substance use disorders being chronic, relapsing conditions and the risks of drug exposure in impoverished communities, participants always ran the risk of drug relapse and medication non-adherence, despite the stern warnings from their trusted physicians. In the words of this participant,

*There’s a word that a lot of us use out in the street. It’s ‘I don’t give a fuck’. It’s something that we use when we’re on this mindset that we just don’t care. A lot of times, I really didn’t care. All I wanted to do was get high and just run the streets, and that’s what I did.*

Yet many of our participants had longstanding HIV infection, and their references to having the same HIV doctor for many years suggested that relationships of mutual trust and respect with clinicians tended to be robust and unconditional.

### Poverty and lack of social capital undermine participants’ engagement in care

Enabling as they were to ART adherence, positive relationships with trusted doctors were nonetheless established within the context of individuals’ experience of the impoverished and resource-poor communities to which they returned, as this participant so cogently summarized:

*But you can't be healthy unless you have all of this. I can't be healthy if I don’t have a place to stay. And I can't be healthy if I'm not eating right. I can't be healthy if I don’t have a job. And I can't be healthy if I don’t have clothes. You got my vicious circle?*

In many participants’ estimations, the *‘world that don’t do drugs’* was where all the medical and economic resources were located, and the participants had to venture there as a condition of their existence. In that process, they felt they encountered discrimination, prejudice, and hostility (whether or not it was ‘objectively’ true) from the criminal justice authorities and other social service agencies and entitlement programs whose task it was to allegedly help them post-incarceration, which contributed to anger, resentfulness, and suspicion. An example of final resignation is shown in the following quote from a participant who perceived obtaining a job post-incarceration as an unattainable dream:

*Nobody’s hiring right now. I wouldn’t care if it was mopping, sweeping the floor right now. NOTHING. If I had a job right now, it would be beautiful. But it's not in the making right now. God didn’t put it in my path, yet.*

Despite this disabling context of poverty and social inequality, clinicians played an important role in enhancing the participants’ perception of their social capital, meaning the inclinations to do things for each other that arise from the collective values of social support (Berkman 2000). As a result, participants expressed a sense of belonging to society from which they had previously felt alienated, and voiced how they wanted to help other people in their communities:

*I'm an addict. I know that if I use, I'm gonna die and I don’t want to die today. I want to be a good mother, a good grandmother. I want to be all I can be. I want to be a drug counselor and I'm gonna do it and I know I'm gonna be good at it, too, because I've been through everything. I've been through the virus. I've been an ex-gang member. I been incarcerated. I been on crack, heroin. I've done it all and I think those people make the best counselors. And I want to work with kids.*

Civic engagement is one of the key elements of social capital (Berkman 2000). Thus the participants’ enthusiasm to put their experiences with addictions, HIV, and criminality to public use through engagement in risk prevention programs may indicate that social capital goes hand in hand with ART adherence, mutually bolstering one another:

*I just love doing things to be a part of stuff. If somebody might write about me, one day, I want to be one of the faces of HIV and AIDS.*

While ‘trust in respected others’ (Altice et al. 2001) and a sense of social belonging stemming from the participants’ positive relationships with their HIV care providers were perceptual categories, they had real and practical consequences for participants, reaffirming their willingness to live by adhering to their appointments and prescribed ART regimens:

*I got this thing: if I go to all my appointments, it’ll give me a reason to live.*

## Discussion

Strategies that effectively improve access to HIV services and adherence to ART are especially crucial for improving health outcomes for HIV-infected PWIDs (Altice et al, 2010), especially those who transition from prisons and jails to communities (Springer et al, 2011). We found that participants’ relationships with family, friends, and clinicians could either facilitate or jeopardize their ART adherence. In all three instances, with family, friends, and clinicians, positive and symmetrical relationships that were based on trust and mutual respect promoted the participants’ sense of social belonging and ability to persevere with ART. Conversely social isolation and ostracism made participants more vulnerable to drug relapse and discontinuing ART. This study contributed to the existing literature (Althoff et al. 2013; Bae et al. 2011) by disentangling how social relationships influence ART adherence. Though others have described individual and healthcare contributors to engaging in HIV generally (Hammett et al. 2015b; Sidibe et al. 2015b; Swan 2015), this study focused on ART adherence and persistence and focused specifically on how supportive and positive social relationships based on mutual respect and trust, including relationships with clinicians in the absence of other kinds of social support, can alleviate feeling alienated from others, which was commonly described by this group of socially marginalized PLH who had transitioned from prison or jail to the community where they lived. This support from ‘trusted others’ provided former prisoners hope, a sense of purpose and social belonging, which are crucial for motivating them to adhere to ART.

Concerning the role of family, our findings echo a recent qualitative study of community-based health care and service professionals that observed that HIV-infected former prisoners often have to work very hard to rebuild relationships with their families upon release, placing HIV care at a markedly lower priority (Sidibe et al., Sidibe et al. 2015a). Positive relationships with family could bolster health-seeking by giving participants a sense of purpose and belonging as well as instrumental support to adhere to ART (Friedman et al. 2007). Yet participants’ family members often either struggled with the same vulnerabilities as the participants themselves, or ostracized participants for fear of recurring economic and social risks stemming from recurrent substance use, and compounded by the combined stigma of HIV, drug-use, and prison experiences, which has been described in research from other countries (Rudolph et al. 2012) and the US related to overlapping stigmas in African American men (Brinkley-Rubinstein 2015). Evidence from outside the US suggests that in the context of prejudice and myths, ostracism of HIV-infected drug-using members is a way of managing secondary HIV- and drug-related stigma directed at families (Salter et al. 2010), and we observed a similar process here.

Concerning relationships with friends, we found that participants who saw themselves interacting with *‘the world that do drugs’* felt alienated from what they called ‘*the world that don’t do drugs’* and perceived mutually respectful friendships bridging the two worlds as unlikely. Instead, recently released PLH who shared common vulnerabilities and experiences became important sources of emotional and instrumental support for each other in managing their HIV and substance use disorders, connected in their close relationships by common experiences and goals. This echoes earlier research that friends and peers can exert positive influences on released prisoners, especially if those influences are harnessed through a health-promoting lens (Broadhead et al., 2002). Relationships with friends, however, may be dangerous when they facilitate relapse to drug use. Back in their resource-poor communities where the drug economy flourished due to poverty and unemployment (Anderson, 2011), we found that participants encountered many untrustworthy former friends they called associates, who reintroduced participants to drug use networks. Close physical proximity to such networks, their omnipresence in resource-poor communities, and multiple connections between participants and network members through shared housing and other scarce resources constituted a ‘risk environment’ (Rhodes et al. 1999) and invoked the social contagion of risky behaviors (Christakis and Fowler 2013), leading to relapse, re-incarceration, and non-adherence to ART.

Concerning relationships with clinicians, we found that participants’ perceptions of themselves as living in a different world from what they called ‘*the world that don’t do drugs*’ were important. Such perceptions followed W.I. Thomas’s (Manis and Meltzer 1967) argument that when people perceive a situation as “real”, regardless of whether it is objectively authentic, it has real consequences for them. As experiences that reinforced their self-perceptions of a lack of credibility in ‘*the world that don’t do drugs*’ accumulated, participants could hit a wall of distrust, in which from a full recovery was seldom perceived as possible (Barbour 1970). Furthermore, reduced physician trust may "go viral" within communities with a large number of former prisoners, spreading to those who have never been to prison themselves (Schnittker 2015). While trust is a fundamental element of social capital that underlies both social support and social participation (Berkman 2000), it was difficult for some participants to trust clinicians, especially if they were not included in decision-making about their HIV treatment. Participants called such clinicians ‘uncaring’ and perceived them as dismissive and patronizing. This finding builds on recent other studies that show that clinicians play an important role in transitional HIV care (Hammett et al. 2015b; Sidibe et al. 2015a, b) and emphasizes that the onus of trust may not be placed solely on ex-prisoners who are often expected to reach out to doctors and activate supportive relationships. Instead, our study suggests that genuine patient centeredness is a core element of the medical system’s mandate, and trust and respect that stems directly from clinicians to ex-prisoners is a key factor enabling medication adherence. This echoes research on asymmetrical power relationships (patients versus clinicians) in the medical system, where ‘acted upon’ patients either reject medicines and treatments outright or accept them after coercion (Embuldeniya et al. 2013; Gibson et al. 2012). Furthermore, some patients perceived discrimination at the hands of their providers for withholding ART from them solely by virtue of their drug use. This perception is reciprocally supported by surveys of HIV experts who would withhold ART if their patient was a PWID (Westergaard et al. 2012). The literature suggests this practice is less common among physicians with more experience treating HIV and working in high drug use prevalence areas (Westergaard et al. 2012). Drug-using patients, however,  are highly sensitive to poor care and may interpret physician’s deferring as a sign of intentional mistreatment (Merrill et al 2002), which could seriously undermine any nascent trust. In cases like our participant who liked his doctor and wished to stop using drugs to ‘please his doctor’ and receive his medication, such a policy could dissuade patients from seeking care during relapse, for fear of disappointing their doctor. It could also reduce medication persistence, or the the time that the patient remains on therapy (Bae et al. 2011), due to the patients’ relapse to drug-use, and the doctors’ suspicion they do so.

Conversely, participants also described genuine patient-centered care by doctors they perceived as caring, whom they felt sincerely respected them as persons and included them in making medical decisions. We found these positive symmetrical relationships when doctors motivated participants to adhere to ART, and gave them a sense of hopefulness for the future. This was described in the earlier literature by the concept of ‘trust in respected others’ (Altice et al. 2001), where instead of reaching down to patients to uplift and improve them, patients and clinicians are seen more as equal partners working together towards a common cause in a mutually supportive and trusting way (Howerton et al. 2007; Wright et al. 2004). We found that even when family support was not forthcoming, a trusting, mutually respectful relationship with their physician gave participants social anchors that inspired them to live and take ART. This finding extends previous research (Odets 1995) on the HIV epidemic in California in the MSM community where marginalized gay men had better care and services once they became diagnosed with HIV. Such positive relationships with their HIV doctors were reported by participants to support them over many years, enduring imminent drug-use relapse, re-incarceration and other chronic risks.

Importantly here, participants were on average 50 years and had known about having HIV for nearly 8 years, which may in part, resulted in them attributing their survival largely to their own resilience and ability to “manage” relationships with individuals and healthcare systems. Future research, however, might examine how relationships with family, friends, and clinicians change over time and how they can differ between those newly diagnosed and long-term survivors. For example, many may have seen friends and peers die from complications of HIV, which could both alter their social networks and influence their own medication-taking behaviors in positive and negative ways. For example, those who had survived for such a long time might have felt impervious to HIV, while others may have felt they did not want to experience the same complications as their peers who had died.

One important finding here is why, if all participants had a SUD, some described positive supportive relationships while others did not. While further inquiry is required, striking differences in experiences of positive support versus no support at all among participants may be grounded in the diversity and heterogeneity of people and their family histories in the communities where poverty is concentrated. For example, why some participants may enjoy more support than others (while having similar personal characteristics and underlying SUDs) may be shaped by the history and patterns of their extended family's geographic and economic mobility, as well as their extended family engagement with the drug economy and the criminal justice system (Anderson 1999). Our participants alluded to these issues when stating their family or old friends now lived out of state.

Our study also had limitations. First, we only focused on the accounts of HIV-infected former prisoners but collected no data from their family, friends, or clinicians. It would be useful in future research studies to triangulate these support structures simultaneously from multiple perspectives. Since social belonging promoted ART adherence but social isolation hindered it, it is important to examine how family, friends, and clinicians reacted to participants’ efforts towards participation in ‘*the world that don’t do drugs*’, and whether this world could let them in. Second, we interviewed former prisoners once within 90 days of their release from prison. While serving time and soon after release, prisoners feel excessive optimism about changing their health behaviors, but readjust their expectations after encountering difficulties of transition like finding housing (Harding et al. 2014). Furthermore, studies elsewhere showed that during and soon after incarceration former prisoners don’t have a realistic sense of their likelihood to relapse to drug use, nursing a false expectation that their addiction is over (Morozova et al. 2013). There would be merit in interviewing HIV-infected prisoners during incarceration and several times after release, to dynamically examine their perspectives regarding social relationships and ART adherence over this period. Third, non-US born former prisoners who have no family in the United States were not captured in our sample. This subset of released prisoners may experience more social isolation than average, furthered by language barriers they may face. Fourth, relationships with peers who are healthcare linkage specialists may also matter to participants’ adherence, but these were not specifically examined in the interviews, and whether such peers may be viewed as friends, or as clinicians. Fifth, for considerations of confidentiality and to ensure that the participants were comfortable sharing their experiences through qualitative interviews, we did not collect the information about their incarceration history or the type of their sentence. While we noted that all participants except one had been incarcerated more than once, there may be potential differences in the quality of social relationships between those incarcerated for short periods of time versus those who were incarcerated for over a year (Meyer et al. 2013) , as well as between men and women. These comparisons constitute areas for further research. Finally, our participants were enrolled in a University-based study where a healthcare provider worked with them over an extended period of time, linking them to care at HIV clinics or other clinics where they received ART. Timing facilitated the building of rapport and bolstered the development of trusting symmetrical relationships. How these experiences may compare to other HIV-infected ex-prisoners needs further research.

## Conclusions

It would be premature to conclude that support from their positive symmetrical relationship with doctors, intact families or trustworthy friends could be sufficient for former prisoners to improve their ART adherence upon release. Our analyses showed that social relationships between recently released PLH with substance use disorders, and their families, friends, and clinicians, occurred in the context of many other unavoidable realities, including persistent unemployment, poverty, the shortage of safe and affordable housing, economic insecurity, and social isolation in resource-poor communities to which participants returned after release (Sledge et al. 2011). The participants invoked the vision of a vicious cycle where they could not see themselves getting healthy unless their other basic needs for food, clothing, shelter, and employment or other meaningful social roles could be met. Thus the impact of social capital on health behaviors must be considered indispensable in connection to inevitable structural inequalities (Friedman et al. 2007).

This leads us to making three arguments for practical implications and improving the status quo. First, our findings underscore the importance of shared decision-making between patient and clinician, and clinicians’ ability to form longstanding, trusting and positive relationship with their HIV patients, which enable ART adherence. Explicitly knowing how much patients value but also depend on them may empower clinicians as well as the patients they serve in a positive feedback loop. Second, participants’ respect and trust in doctors suggests that further bundling clinical services with social services, such as assistance with employment, housing and social security, may be effective. Mistrust of some participants towards the medical system stemmed from their overall experiences of alienation from the wider society and its institutions (Comfort, 2008). Feeling genuine respect and being taken seriously by a doctor was significant and generated reciprocal trust. Building on this fledgling mutual trust, collaboration among all the stakeholders of the process—namely, participants, clinicians, employers, welfare officers, and prison custodial staff—may be a vehicle for positive social change. Third, while our participants tried staying away from former friends and environments associated with drug use to avoid relapse, which echoes research on Alcoholics and Narcotics Anonymous mutual help organizations (Polcin et al. 2010), our findings suggest that discharge planning programs need to support participants in the community and promote positive social activities that will counterbalance negative forces. One example of such an activity may be participation in clinical studies such as this one (corroborating previous research that HIV-care providers affiliated with academic institutions facilitate the effective transition of HIV-infected prisoners to the community through patient-centeredness and personal connection with the patients) (Hammett et al. 2015a). Indeed, participants appreciated that stories of their experiences were heard and valuable, which enhanced their sense of belonging, trust, and reciprocity, even if temporarily. Our study affirmed to participants they could offer to ‘*the world that don’t do drugs*’ a wealth of knowledge and experience, even inspired some participants to offer to play the role of the interviewers in similar studies, and to inquire about counselors and outreach workers employment positions. If hired as peer advocates participants like ours can assist newly-released HIV-positive prisoners to navigate the exigencies of healthcare and social recovery.

The syndemic of HIV, drug use, and incarceration is exacerbated by the negative and marginalizing factors of poverty, stigma, and social isolation. Yet we hope that giving voice to individuals affected by HIV and involving them throughout the research process helps to bridge PLH involved in the criminal justice system to the community and to facilitate a positive transition back into society.
